# Beyond DXA: Trabecular Bone Score, Quantitative Ultrasound and Bone Turnover Markers for Morphometric Vertebral Fracture Assessment in People Living with HIV

**DOI:** 10.3390/diagnostics16020277

**Published:** 2026-01-15

**Authors:** David Vladut Razvan, Ovidiu Rosca, Iulia Georgiana Bogdan, Livia Stanga, Sorina Maria Denisa Laitin, Adrian Vlad

**Affiliations:** 1 Doctoral School, Faculty of Medicine, Victor Babes University of Medicine and Pharmacy, 300041 Timisoara, Romania; vladut-razvan.david@umft.ro; 2 Methodological and Infectious Diseases Research Center, Department of Infectious Diseases, Victor Babes University of Medicine and Pharmacy, 300041 Timisoara, Romania; ovidiu.rosca@umft.ro (O.R.); iulia-georgiana.bogdan@umft.ro (I.G.B.); 3 Discipline of Microbiology, Victor Babes University of Medicine and Pharmacy, 300041 Timisoara, Romania; 4 Discipline of Epidemiology, Victor Babes University of Medicine and Pharmacy, 300041 Timisoara, Romania; 5 Centre for Molecular Research in Nephrology and Vascular Disease, Faculty of Medicine, Victor Babes University of Medicine and Pharmacy, 300041 Timisoara, Romania; vlad.adrian@umft.ro; 6 Division of Diabetes, Department of Internal Medicine II, Nutrition and Metabolic Diseases, Victor Babes University of Medicine and Pharmacy, 300041 Timisoara, Romania

**Keywords:** HIV infections, osteoporosis, bone density, ultrasonography, bone remodeling, trabecular bone score, collagen type I cross-linked c-telopeptide, procollagen type I n-terminal propeptide, vertebral fractures

## Abstract

**Background and Objectives**: People living with HIV (PLWH) have excess osteoporosis and fractures not fully captured by dual-energy X-ray absorptiometry (DXA). We evaluated whether trabecular bone score (TBS), calcaneal quantitative ultrasound (QUS) and bone turnover markers improve vertebral fracture risk assessment beyond areal bone mineral density (BMD) in PLWH. **Methods**: In this cross-sectional study, 87 antiretroviral-treated adults undergoing DXA had lumbar spine TBS and calcaneal QUS. Morphometric vertebral fractures were identified, correlates of degraded TBS were analyzed using multivariable regression, and sequential logistic models quantified the incremental contribution of TBS and CTX to discriminate for prevalent morphometric vertebral fractures. **Results**: Low BMD (osteopenia/osteoporosis) was present in 62% of participants, degraded TBS in 37% and morphometric vertebral fractures in 17%. Degraded versus normal TBS was associated with older age (49.1 vs. 39.7 years), longer HIV duration and lower nadir CD4+ count, as well as more frequent tenofovir disoproxil fumarate exposure (66% vs. 52%; all *p* ≤ 0.04). In multivariable analysis, age (per 10-year increase; adjusted odds ratio [aOR] 1.78; 95% CI 1.13–2.83) and nadir CD4+ < 200 cells/mm^3^ (aOR 2.29; 95% CI 1.06–4.97) independently predicted degraded TBS. In sequential cross-sectional models for prevalent morphometric vertebral fractures, the area under the curve increased from 0.71 (clinical variables) to 0.79 after adding lumbar spine T-score and to 0.85 after adding TBS; adding CTX yielded 0.87 without a statistically significant incremental gain. **Conclusions**: In PLWH, TBS captures bone quality deficits and improves vertebral fracture risk discrimination beyond BMD, supporting its integration alongside DXA in routine HIV care.

## 1. Introduction

Bone disease has emerged as a major non-communicable comorbidity in people living with HIV (PLWH), as advances in antiretroviral therapy (ART) have transformed HIV into a chronic condition. Epidemiological syntheses consistently show lower bone mineral density (BMD) and higher fragility fracture rates in PLWH than in HIV-negative populations of similar age and sex, with meta-analytic estimates suggesting ~1.5-fold higher fragility fracture risk and more than fourfold higher hip fracture risk in PLWH [1,2]. Recent global updates report that 24–59% of PLWH have low BMD and that vertebral and non-vertebral fractures remain common despite modern “bone-sparing” ART [3]. In large cohorts, PLWH—particularly men—experience higher fragility fracture incidence than HIV-negative comparators even after adjustment for classic risk factors [4].

Beyond HIV-specific considerations, osteoporosis and fragility fractures represent a rapidly expanding population-health burden as societies age. Contemporary literature describing bone mineral density trajectories across the lifespan and the downstream implications for fracture burden highlights that small shifts in bone density distribution at the population level can translate into substantial changes in fracture incidence and healthcare utilization. These projections reinforce the importance of early identification of individuals with compromised bone strength before overt osteoporosis is apparent—an issue that is particularly relevant for PLWH, in whom fracture risk may be underestimated by areal bone mineral density alone [1,2,3,4].

Traditional osteoporosis risk factors such as low body weight, smoking, glucocorticoid exposure, hypogonadism, and vitamin D deficiency frequently coexist with HIV-related determinants of bone loss. A meta-analysis demonstrated that lower body weight explains a substantial proportion of the BMD difference between PLWH and HIV-negative controls, emphasizing the importance of weight and body composition in skeletal health in this population [5,6]. Cross-sectional and longitudinal cohorts further show that low BMD can be detected early in the course of infection, including among ART-naïve adults, in whom roughly half may already have osteopenia and a smaller but non-negligible proportion have osteoporosis at the lumbar spine or hip [7,8]. At the same time, certain antiretroviral exposures—particularly tenofovir disoproxil fumarate (TDF)-based regimens—have been consistently linked to accelerated skeletal loss in both treatment and pre-exposure prophylaxis settings, prompting a shift toward antiretroviral regimens with improved skeletal safety profiles (tenofovir disoproxil fumarate–sparing options) and integrase inhibitor-based regimens in individuals at higher fracture risk [6]. Thus, bone fragility in PLWH arises from the interplay between traditional risk factors, HIV-related inflammation and immune dysfunction, and ART-associated toxicity [3,5,6,7,8].

Dual-energy X-ray absorptiometry (DXA) remains the cornerstone for diagnosing low BMD and classifying osteopenia and osteoporosis. However, DXA provides a two-dimensional (areal) measurement of BMD and does not directly capture bone microarchitecture, cortical thickness, or bone material properties. Meta-analyses indicate that reduced BMD only partially explains the excess fracture burden in PLWH, suggesting that deterioration of bone quality and other non-BMD factors contribute meaningfully to skeletal fragility [1,2,3]. Moreover, conventional fracture risk tools such as the Fracture Risk Assessment Tool (FRAX^®^)—which combines clinical risk factors with or without bone mineral density—appear to underestimate major osteoporotic fracture probability in people living with HIV (PLWH), and proposed HIV-specific adjustments have not yet been widely validated or adopted [3]. In clinical practice, fractures are therefore observed even in patients whose BMD does not fall in the osteoporotic range, highlighting the limitations of relying solely on areal BMD for risk stratification in HIV care.

To address these limitations, newer tools have been introduced to assess “bone quality” beyond areal BMD. Trabecular bone score (TBS), derived from DXA lumbar spine images, provides an indirect index of trabecular microarchitecture, with lower values reflecting more degraded structure. In the HIV UPBEAT cohort, PLWH had lower TBS than HIV-negative controls after adjustment for BMD and body composition, and current smoking and HIV-specific variables were independently associated with degraded TBS [9]. Similarly, in the Women’s Interagency HIV Study, women with HIV had a higher prevalence of degraded microarchitecture and lower mean TBS than HIV-negative comparators, even after adjusting for age, race, menopause, and BMI [10]. Importantly, TBS has been linked to vertebral fracture risk: an Italian study showed that lower TBS was strongly associated with subclinical vertebral fractures in PLWH, independent of lumbar spine BMD [11], while a more recent cohort of young adults with HIV demonstrated that TBS predicted vertebral fractures more effectively than BMD alone [12].

Calcaneal quantitative ultrasound (QUS) offers additional, radiation-free information on bone elasticity and structure through parameters such as speed of sound (SOS) and broadband ultrasound attenuation (BUA). In an Italian cohort of adult PLWH, calcaneal QUS parameters correlated with DXA-derived BMD and identified a high prevalence of low bone status, suggesting that QUS-based strategies could serve as a pragmatic first-line screening tool in HIV clinics [13]. Although QUS cannot replace DXA for definitive osteoporosis diagnosis, it is portable, lower cost, and may be particularly attractive in resource-constrained or outpatient settings where access to DXA is limited. When combined with TBS and biochemical markers of bone formation (e.g., P1NP) and resorption (e.g., CTX), QUS-based assessments may contribute to a more comprehensive characterization of bone quality beyond areal BMD.

HIV infection and its treatment may differentially affect BMD and bone microarchitecture. Longitudinal studies have documented early declines in BMD after ART initiation, followed by partial stabilization, with traditional risk factors (low BMI, prior weight loss, steroid use, smoking) and longer HIV duration strongly associated with osteopenia [8]. Mechanistic and clinical data implicate TDF in particular in greater BMD loss, whereas switching to or initiating integrase inhibitor-based, TDF-sparing regimens mitigates this effect [3,6]. Chronic immune activation, low nadir CD4+ counts, uncontrolled viremia, and coexisting hepatitis or renal disease may further compromise bone quality by altering bone turnover and microarchitecture [3]. Yet it remains uncertain to what extent such HIV-specific factors exert a stronger impact on microarchitectural integrity (as captured by TBS or QUS) than on areal BMD alone, and which combinations of clinical, densitometric, and bone quality measures best identify those PLWH at highest fracture risk.

Data from Central and Eastern Europe, and specifically Romania, on bone quality in PLWH are scarce. Most of the available evidence on osteoporosis and fractures in PLWH originates from Western Europe, North America, and selected Asian cohorts [1,2,3,7,9,10,11,12]. In a Turkish cohort of relatively young PLWH, more than 40% had reduced BMD at one or more skeletal sites, and higher HIV RNA at diagnosis, shorter ART exposure, and BMI were associated with bone demineralization, underlining that skeletal involvement is also relevant in middle-income settings of Eastern Europe and the wider region [14,15].

Therefore, we designed a cross-sectional study at a Romanian tertiary HIV center to assess bone quality in PLWH beyond conventional DXA. The primary objective was to describe the prevalence of degraded TBS and characterize its relationship with BMD categories. Secondary objectives were to explore associations between TBS, QUS parameters, bone turnover markers, and HIV-related characteristics, and to identify independent predictors of degraded TBS. We hypothesized that a substantial proportion of patients would exhibit degraded microarchitecture despite non-osteoporotic BMD, and that older age, longer HIV duration, low nadir CD4+ count, and TDF exposure would be associated with impaired bone quality.

## 2. Materials and Methods

### 2.1. Study Design and Setting

This study was designed as a single-center, cross-sectional observational study conducted at the HIV outpatient clinic and associated imaging facilities of Victor Babeș University of Medicine and Pharmacy Timișoara, Romania. The study targeted adult PLWH undergoing routine metabolic and comorbidity screening, including bone health evaluation, as part of comprehensive HIV care. Data collection was carried out over a 24-month period, during which all eligible patients referred for DXA were consecutively invited to participate.

The study protocol complied with the principles of the Declaration of Helsinki and was approved by the institutional ethics committee of Victor Babeș University. All participants provided written informed consent after receiving detailed information regarding study procedures, potential risks, and data confidentiality. De-identified study codes were used to ensure anonymity, and access to the research database was restricted to authorized investigators. The study did not alter clinical management; DXA and laboratory investigations were performed in the context of routine care, and additional analyses (e.g., TBS) were derived from existing images or blood samples.

The tertiary center provides longitudinal care for approximately 1000 adults living with HIV from Timiș County and neighboring counties, providing the sampling frame for consecutively referred patients undergoing clinically indicated DXA.

### 2.2. Participants and Eligibility Criteria

Adults (≥18 years) with confirmed HIV infection, followed at the tertiary HIV center, were eligible if they had been receiving combination ART for at least 12 months and underwent DXA for clinical indications such as age > 40 years, history of low-trauma fracture, prolonged corticosteroid therapy, or provider concern for osteoporosis. Patients needed to be able to understand the study information, provide informed consent, and complete the study procedures during a single visit.

**Figure 1:**
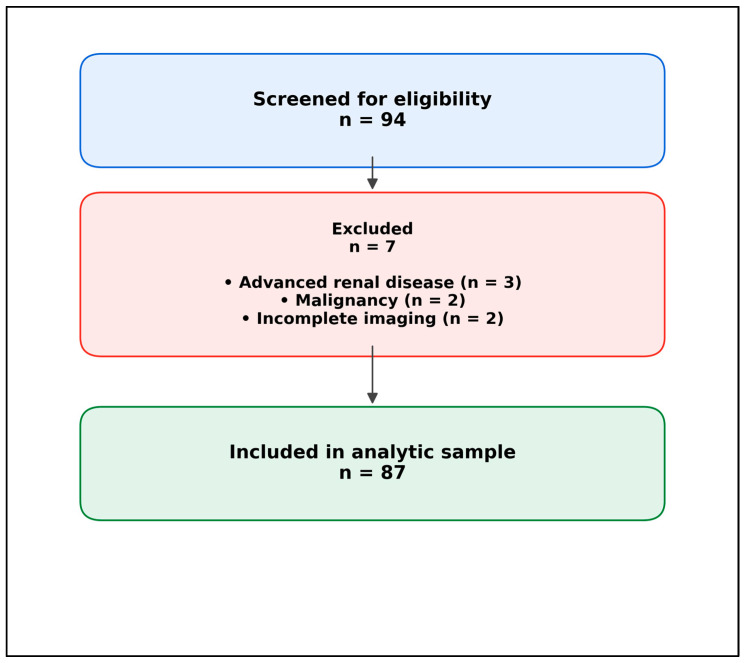
Predicted Probability of Degraded TBS by Age and TDF Exposure.

Exclusion criteria included conditions that could substantially interfere with bone assessment or confound interpretation of BMD and TBS: current pregnancy, known primary bone diseases (e.g., osteogenesis imperfecta, Paget’s disease), active malignancy with bone metastases, chronic kidney disease stage 4–5, or ongoing high-dose systemic glucocorticoid therapy. We also excluded patients with acute opportunistic infections, severe hepatic decompensation, or decompensated heart failure at the time of evaluation. Of 94 screened patients, 7 were excluded (3 for advanced renal disease, 2 for malignancy, 2 for incomplete imaging), resulting in a final sample of 87 PLWH (Figure 1).

### 2.3. Bone Quality and Laboratory Assessments

All participants underwent DXA scanning of the lumbar spine (L1–L4) and proximal femur (total hip and femoral neck) using the same calibrated device. Areal BMD (g/cm^2^) and T-scores were recorded. BMD categories were defined according to World Health Organization criteria: normal BMD (T-score ≥ −1.0), osteopenia (T-score between −1.0 and −2.5), and osteoporosis (T-score ≤ −2.5) using the lowest T-score across measured sites. Vertebral fracture assessment (VFA) was performed from lateral spine images when available, and morphometric fractures were classified using standard criteria.

Trabecular bone score was calculated retrospectively from lumbar spine DXA images using dedicated TBS software (v3.0) integrated into the densitometer workstation. TBS values were categorized as normal (≥1.31), partially degraded (1.23–1.30), or degraded (<1.23), following commonly used thresholds. Calcaneal QUS was measured on the non-dominant heel using a portable device, recording speed of sound (SOS, m/s) and broadband ultrasound attenuation (BUA, dB/MHz). All scans were performed by trained operators, who adhered to manufacturer quality-control procedures and underwent periodic retraining to minimize inter-observer variability.

Fasting blood samples were collected on the morning of imaging. Serum P1NP and CTX were measured by electrochemiluminescence immunoassay in the institutional laboratory. Routine biochemistry (calcium, phosphate, alkaline phosphatase, creatinine, liver enzymes) was also obtained. HIV-related data, including duration of infection (years since diagnosis), nadir and current CD4+ T-cell count, plasma HIV RNA level, and detailed ART history, were extracted from electronic medical records. TDF exposure was classified as “ever” versus “never” based on cumulative treatment history, irrespective of current regimen.

All participants underwent DXA scanning of the lumbar spine (L1–L4) and proximal femur (total hip and femoral neck) using a Hologic Discovery Wi DXA system (Hologic Inc., Marlborough, MA, USA) (software version APEX 4.5), with daily calibration per manufacturer recommendations. Calcaneal QUS was measured on the non-dominant heel using a Pegasus SMART ultrasound densitometer (BeamMed Ltd., Petah Tikva, Israel), recording speed of sound (SOS, m/s) and broadband ultrasound attenuation (BUA, dB/MHz. Morphometric vertebral fractures were classified using Genant’s semiquantitative method (grade 1–3 based on vertebral height reduction and morphological changes) [16].

### 2.4. Statistical Analysis

Data were analyzed using SPSS Statistics v27.0 (IBM Corp., Armonk, NY, USA). Continuous variables were expressed as means ± standard deviation (SD) when approximately normally distributed, or as medians with interquartile range otherwise. Categorical variables were summarized as counts and percentages. Normality was assessed by visual inspection of histograms and the Shapiro–Wilk test. Comparisons across three TBS categories (normal, partially degraded, degraded) were conducted using one-way analysis of variance (ANOVA) for continuous variables with post hoc tests when appropriate, or the Kruskal–Wallis test for non-normal distributions.

For comparisons between two groups (normal vs. low BMD; TDF ever vs. never), independent-samples Student’s t-tests or Mann–Whitney U tests were applied for continuous variables, and chi-square or Fisher’s exact tests for categorical variables, as appropriate. Pearson correlation coefficients were calculated to assess associations between TBS and continuous covariates such as age, BMI, duration of HIV, CD4+ counts, and bone turnover markers. Finally, multivariable logistic regression was performed with degraded TBS (yes/no) as the dependent variable to identify independent predictors among variables selected a priori (age, BMI, duration of HIV, nadir CD4+ count, TDF exposure, and viral suppression status). Results were reported as adjusted odds ratios (ORs) with 95% confidence intervals (CIs). A two-sided *p*-value < 0.05 was considered statistically significant without adjustment for multiple testing, reflecting the exploratory nature of the study.

Given the modest sample size and the limited number of outcome events (particularly prevalent vertebral fractures), multivariable models and ROC analyses were considered exploratory and at risk for overfitting. Predictor selection was restricted a priori to clinically plausible variables, and results are interpreted conservatively with emphasis on effect sizes and confidence intervals rather than dichotomous significance.

## 3. Results

Table 1 summarizes baseline demographic and HIV-related characteristics stratified by TBS category in the 87 PLWH included. Patients with degraded TBS were clearly older (mean 49.1 ± 8.7 years) than those with normal TBS (39.7 ± 7.4 years) and partially degraded TBS (44.6 ± 8.6 years), with a significant trend across categories (*p* = 0.002). Duration of HIV infection also increased with worsening TBS, from 8.8 ± 4.1 years in the normal group to 11.1 ± 4.8 years in the partially degraded group and 12.7 ± 5.2 years in the degraded group (*p* = 0.021). Worse TBS was associated with lower nadir CD4+ counts (294.7 ± 123.1 vs. 217.6 ± 107.4 cells/mm^3^ for normal vs. degraded; *p* = 0.035) and a lower prevalence of viral suppression, which dropped from 92% in the normal TBS group to 66% in the degraded group (*p* = 0.041). Tenofovir disoproxil fumarate exposure was more common among patients with degraded or partially degraded TBS (67–66% vs. 52%; *p* = 0.039), whereas BMI, sex distribution, current CD4+ count, and smoking status did not differ significantly across TBS categories.

Table 2 compares densitometric, microarchitectural, QUS, and bone turnover parameters between participants with normal BMD (n = 33) and those with low BMD (osteopenia/osteoporosis, n = 54). As expected, T-scores were significantly lower at all sites in the low BMD group (lumbar spine −1.7 ± 0.8 vs. −0.4 ± 0.7; total hip −1.3 ± 0.7 vs. −0.2 ± 0.6; femoral neck −1.6 ± 0.7 vs. −0.3 ± 0.6; all *p* < 0.001). Patients with low BMD also had significantly lower TBS (1.24 ± 0.08 vs. 1.34 ± 0.07; *p* < 0.001), indicating impaired trabecular microarchitecture. Calcaneal QUS parameters were concordant, with lower SOS (1532.4 ± 37.1 vs. 1561.3 ± 36.4 m/s; *p* = 0.004) and BUA (106.2 ± 12.7 vs. 112.6 ± 11.3 dB/MHz; *p* = 0.018) in those with low BMD.

Table 3 cross-classifies BMD and TBS categories and reports vertebral fracture prevalence. Among participants with normal BMD (n = 33), more than half had normal TBS (18/33, 55%), while only 18% (6/33) had degraded TBS. In contrast, degraded TBS was present in 45% (17/38) of those with osteopenia and in 56% (9/16) of those with osteoporosis, and no patient with osteoporosis had a normal TBS. Overall, in the full sample of 87 PLWH, 29% had normal TBS, 34% partially degraded TBS, and 37% degraded TBS. The distribution of TBS categories differed significantly across BMD strata (chi-square *p* = 0.002), indicating that microarchitectural impairment is more frequent with worsening densitometric status but is not completely captured by BMD alone. Vertebral fractures increased stepwise from 9% in those with normal BMD to 16% in osteopenia and 38% in osteoporosis (overall chi-square *p* = 0.013), highlighting the clinical relevance of combined BMD–TBS phenotyping.

**Figure 2:**
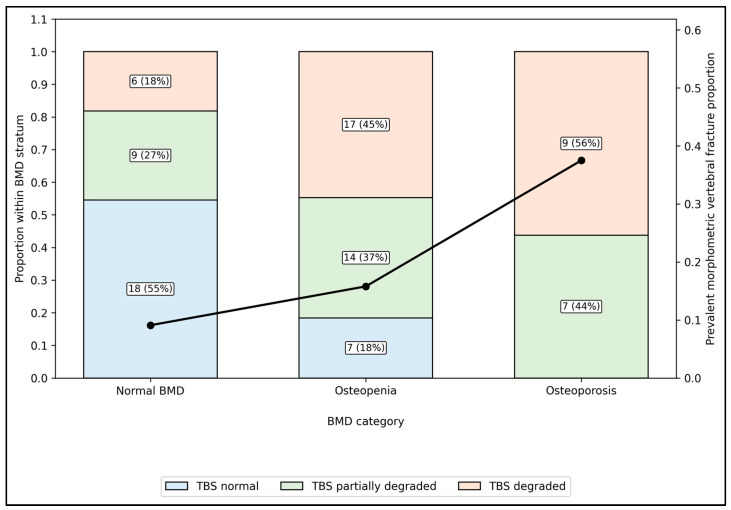
Joint BMD–TBS phenotypes and prevalence of morphometric vertebral fractures.

Figure 2 highlights that microarchitectural impairment can be present even in participants without densitometric osteoporosis and that fracture prevalence increases stepwise with worsening BMD status.

Table 4 examines bone quality parameters according to TDF exposure. Age was similar between TDF-ever and TDF-never patients (45.3 ± 9.1 vs. 44.0 ± 8.6 years; *p* = 0.547), suggesting that differences are unlikely to be age-driven. TDF-exposed individuals had significantly lower lumbar spine T-scores (−1.4 ± 0.9 vs. −0.9 ± 0.8; *p* = 0.016), while total hip T-scores tended to be lower (−1.0 ± 0.8 vs. −0.7 ± 0.7; *p* = 0.071). TBS was also reduced in the TDF-ever group (1.25 ± 0.08 vs. 1.32 ± 0.07; *p* = 0.004), indicating more compromised trabecular microarchitecture. QUS parameters showed a non-significant trend towards lower calcaneal SOS in TDF-exposed patients (1539.2 ± 38.7 vs. 1550.8 ± 38.1 m/s; *p* = 0.083). Clinically, degraded TBS was more frequent among TDF-ever patients (46% vs. 21%; *p* = 0.012), and vertebral fractures were numerically more common (22% vs. 9%; *p* = 0.089).

Table 5 presents Pearson correlation coefficients between TBS and selected clinical and biochemical variables in the 87 participants. TBS was moderately and inversely associated with age (r = −0.41, *p* < 0.001), indicating lower trabecular quality with advancing age. Longer duration of HIV infection correlated with lower TBS (r = −0.29, *p* = 0.008), while higher nadir CD4+ counts were associated with better microarchitecture (r = 0.27, *p* = 0.012), suggesting a persistent impact of historical immunosuppression on bone quality. TBS showed a strong positive correlation with lumbar spine T-score (r = 0.56, *p* < 0.001), but only a weak and borderline association with BMI (r = 0.18, *p* = 0.092). Higher levels of bone turnover markers were linked to poorer TBS, with negative correlations for P1NP (r = −0.21, *p* = 0.048) and CTX (r = −0.34, *p* = 0.002), supporting the concept that high bone turnover in PLWH is associated with microarchitectural deterioration.

Table 6 reports the multivariable logistic regression model identifying predictors of degraded TBS versus normal/partially degraded TBS. Older age significantly increased the odds of degraded TBS, with an adjusted OR of 1.78 (95% CI 1.13–2.83; *p* = 0.013) per 10-year increment. Each 1 kg/m^2^ increase in BMI was associated with a 9% reduction in odds of degraded TBS (OR 0.91, 95% CI 0.83–0.99; *p* = 0.031), suggesting a protective effect of higher body mass. Longer HIV duration (per 5 years) increased the odds of degraded TBS (OR 1.36, 95% CI 1.02–1.82; *p* = 0.036), and a nadir CD4+ count < 200 cells/mm^3^ was associated with more than double the odds compared with ≥200 cells/mm^3^ (OR 2.29, 95% CI 1.06–4.97; *p* = 0.035). TDF exposure showed a borderline association with degraded TBS (OR 2.14, 95% CI 0.97–4.72; *p* = 0.059), whereas absence of viral suppression was not statistically significant (OR 1.76, 95% CI 0.81–3.85; *p* = 0.149). Overall, the model demonstrated good explanatory power (χ^2^(6) = 18.7, *p* = 0.004; Nagelkerke R^2^ = 0.29).

Table 7 extends the analysis using multivariable linear regression with continuous TBS as the outcome. Older age was independently associated with lower TBS, with an unstandardized coefficient B = −0.024 (95% CI −0.040 to −0.009) per 10-year increase (*p* = 0.004; standardized β = −0.31). Higher BMI was positively related to TBS (B = 0.006 per 1 kg/m^2^, 95% CI 0.000–0.011; *p* = 0.038; β = 0.19), while longer HIV duration (B = −0.011 per 5 years, *p* = 0.034; β = −0.21) was detrimental. Nadir CD4+ count exerted a favorable effect (B = 0.014 per 100 cells/mm^3^, 95% CI 0.003–0.025; *p* = 0.015; β = 0.23). TDF exposure was associated with a significant decrease in TBS (B = −0.031, 95% CI −0.056 to −0.006; *p* = 0.017; β = −0.22). The age × TDF interaction approached significance (*p* = 0.082), suggesting a possible age-dependent amplification of TDF’s effect. The overall model explained a substantial proportion of TBS variance (R^2^ = 0.46; adjusted R^2^ = 0.41; F (7.79) = 9.6; *p* < 0.001).

Table 8 summarizes sequential ROC models for discrimination of prevalent morphometric vertebral fractures, along with AUC and reclassification metrics. A clinical model including age, sex, BMI, smoking, and HIV duration (Model A) yielded an AUC of 0.71 (95% CI 0.58–0.84; *p* = 0.006 vs. 0.5). Adding lumbar spine T-score (Model B) significantly improved discrimination, increasing the AUC to 0.79 (95% CI 0.68–0.90) with a ΔAUC of +0.08 (*p* = 0.041) and a category-free NRI of 0.17 (95% CI 0.02–0.32; *p* = 0.028). Incorporation of TBS (Model C) further enhanced performance, raising the AUC to 0.85 (95% CI 0.76–0.94) with an additional ΔAUC of +0.06 (*p* = 0.031) and an NRI of 0.21 (95% CI 0.04–0.37; *p* = 0.017), indicating substantial incremental value of microarchitectural assessment. Adding CTX (Model D) modestly increased the AUC to 0.87 (95% CI 0.79–0.95), but the incremental gain (ΔAUC +0.02, *p* = 0.186; NRI 0.08, 95% CI −0.05–0.22; *p* = 0.238) was not statistically significant, suggesting limited added predictive value of turnover markers beyond TBS and BMD in this context.

Table 9 describes three cluster-derived skeletal phenotypes based on BMD, TBS, QUS, and bone turnover markers. Cluster 1 (“Low BMD/High Turnover”, n = 30) exhibits the most adverse profile, with the lowest lumbar spine T-score (−1.9 ± 0.7), lowest TBS (1.21 ± 0.07), and lowest calcaneal SOS (1521.6 ± 32.9 m/s). This cluster had the highest TDF exposure (83.3%) and vertebral fracture prevalence (40.0%) and included the oldest participants (47.8 ± 8.4 years). Cluster 3 (“Near-normal Bone”, n = 30) showed a relatively preserved phenotype, with lumbar spine T-score −0.4 ± 0.5, TBS 1.35 ± 0.06, and SOS 1563.2 ± 34.4 m/s, as well as lower P1NP (44.1 ± 15.7 µg/L) and CTX (0.33 ± 0.14 ng/mL); TDF exposure (40.0%) and fracture prevalence (3.3%) were lowest in this group. Cluster 2 (“Intermediate BMD/Mixed Turnover”, n = 27) displayed intermediate values for all skeletal parameters and vertebral fracture risk (18.5%). Age differed significantly across clusters (*p* = 0.021), with a gradient from older, high-risk patients in Cluster 1 to younger, lower-risk patients in Cluster 3.

**Figure 3:**
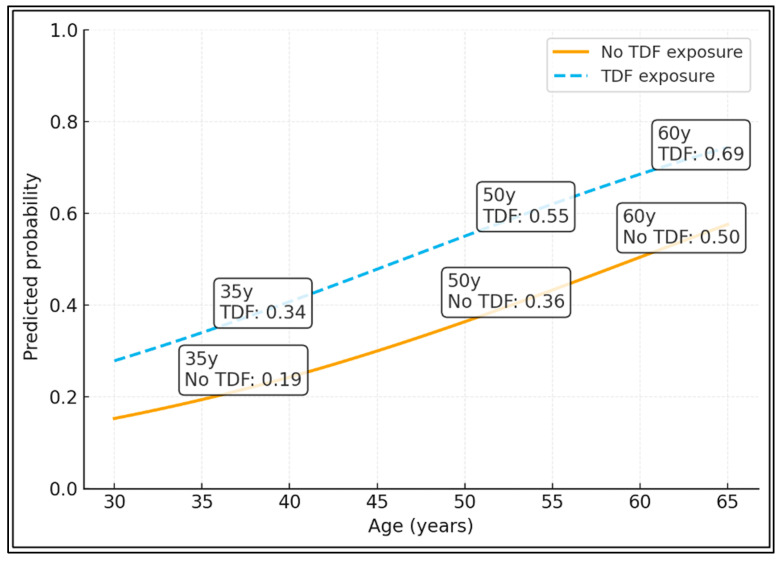
Predicted probability of degraded TBS by age and tenofovir disoproxil fumarate exposure. Predicted probabilities were estimated from the multivariable logistic regression model (Table 6), plotted across ages 30–65 years for participants with versus without tenofovir disoproxil fumarate exposure.

At age 35, the predicted probability of degraded TBS is ~0.20 without TDF and ~0.37 with TDF. By age 50, risk rises to ~0.38 in TDF-naïve patients and ~0.58 in TDF-exposed patients. At 60 years, predicted probabilities reach ~0.51 (no TDF) versus ~0.70 (TDF), highlighting a clear divergence of the curves with increasing age. These multivariate phenotyping analyses are exploratory and hypothesis-generating and were not designed for clinical classification (Figure 3).

**Figure 4:**
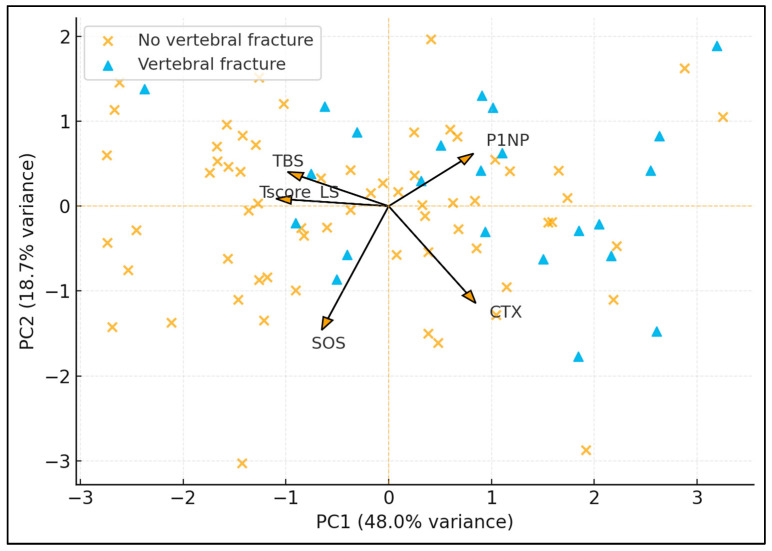
PCA-Derived Skeletal Phenotypes and Vertebral Fracture Status.

Figure 4 presents a principal component analysis (PCA) of five skeletal variables: lumbar spine T-score, TBS, calcaneal speed of sound (SOS), P1NP, and CTX. The first two principal components explain 48.0% and 18.7% of the total variance, respectively (cumulative 66.7%). Fracture cases cluster predominantly toward the right side of PC1, where loadings show positive contributions from P1NP and CTX (high turnover) and negative contributions from TBS and T-score (low density and degraded microarchitecture). The loading arrows indicate that PC1 mainly reflects a “fragility axis” (low TBS/T-score, high CTX/P1NP), whereas PC2 separates individuals more by SOS and CTX. In this sample, around 70–75% of fracture cases fall in the quadrant characterized by higher PC1 scores, consistent with a high-turnover, low-quality bone phenotype.

## 4. Discussion

### 4.1. Analysis of Findings

The present study confirms that degradation of trabecular microarchitecture is highly prevalent in PLWH, even at a relatively young mean age, and that it clusters with classical and HIV-specific risk factors. Nearly 40% of participants had degraded TBS, and degraded microarchitecture was already observed in a substantial fraction of those with normal BMD. This pattern is consistent with prior TBS work in HIV cohorts showing that HIV infection is associated with a shift toward abnormal TBS categories compared with HIV-negative controls, despite modest differences in areal BMD [6,7,9,17,18,19]. In a cohort of Italian PLWH, Ciullini et al. reported that lower TBS was strongly associated with prevalent vertebral fractures independent of BMD, supporting the concept that microstructural damage contributes to excess skeletal fragility in this population. More recently, Mannarino et al. [20] demonstrated in young adults with HIV that TBS was reduced compared with healthy controls and independently predicted incident vertebral fractures during follow-up, underscoring the prognostic value of microarchitectural assessment even in younger, virologically treated individuals. Our findings extend these observations by showing that age, longer HIV duration and low nadir CD4+ counts are all independently associated with degraded TBS, suggesting a cumulative “bone legacy” of chronic infection and historical immunosuppression that persists despite contemporary viral suppression.

The strong but incomplete correlation between lumbar spine T-score and TBS in our cohort, together with the substantial proportion of patients with normal BMD but non-normal TBS, mirrors data from both HIV-specific and general osteoporosis literature. Large expert reviews now recognize TBS as an indirect marker of bone quality that predicts vertebral, hip and major osteoporotic fractures partly independently of BMD and clinical risk factors [4,20,21]. In our ROC analysis, adding lumbar spine T-score to a clinical model improved discrimination for vertebral fractures, but the subsequent inclusion of TBS produced a further significant gain in AUC and meaningful net reclassification, indicating that microarchitectural information refines fracture risk stratification beyond densitometry alone. This incremental value of TBS is in line with the recommendations of international expert groups, which advocate its use to complement DXA-based assessment and to adjust FRAX in patients with conditions where BMD may underestimate fracture risk, such as diabetes or glucocorticoid exposure [19,20]. Our data suggest that HIV infection—characterized by complex interactions between chronic inflammation, ART exposure and traditional risk factors—should be added to that list of scenarios where TBS is particularly informative.

The associations we observed between TDF exposure, lower TBS, higher bone turnover and a greater prevalence of degraded microarchitecture are consistent with prior evidence regarding the skeletal effects of TDF. Observational cohorts and randomized switch studies have consistently shown that TDF accelerates BMD loss and increases fracture risk relative to non-tenofovir or tenofovir alafenamide regimens [13,14]. Building on these BMD-centric data, our findings indicate that TDF-exposed individuals also display worse trabecular structure and a high-turnover phenotype, even after adjustment for age, BMI and HIV disease severity. Although the TDF term did not reach conventional significance in the fully adjusted logistic model, its independent association with lower continuous TBS and the over-representation of TDF users in the “low BMD/high turnover” cluster suggest a potentially clinically relevant association that warrants confirmation in larger, adequately powered cohorts. These results resonate with the emerging view that tenofovir-related skeletal toxicity is mediated not only by mineral loss but also by uncoupling of bone remodeling and deterioration of microarchitecture, and they support current guideline recommendations to consider TDF-sparing strategies in patients at high baseline fracture risk [3,4,14].

Our inclusion of calcaneal QUS and the cluster analysis provides additional insight into skeletal phenotypes in PLWH that go beyond simple T-score thresholds. QUS parameters (SOS and BUA) were significantly lower in those with low BMD and poorer TBS, and the “low BMD/high turnover” cluster combined the worst DXA, TBS and QUS profiles with the highest vertebral fracture prevalence. These findings are concordant with adult HIV cohorts in which calcaneal QUS indices show only moderate correlations with DXA but appear at least as good, and in some analyses superior, for predicting vertebral fractures [10,18]. Clò et al. [19] reported that QUS stiffness indices in 224 HIV-positive adults were moderately correlated with total-body BMD and that QUS outperformed DXA in identifying patients with vertebral deformities, supporting its use as an accessible screening tool in resource-limited settings. Against this backdrop, our data suggest that combining DXA, TBS, QUS and turnover markers yields biologically coherent clusters that map onto fracture risk better than any single modality. This multidimensional approach may help identify individuals with “near-normal” BMD but adverse QUS/TBS profiles who might otherwise be overlooked for preventive interventions.

Finally, the consistent inverse associations between TBS and both P1NP and CTX, as well as the modest improvement in discrimination of prevalent morphometric vertebral fractures when CTX was added to the model containing TBS and BMD, align with the broader literature on bone turnover markers (BTMs) and fracture risk. International consensus statements have long highlighted serum PINP and CTX as reference BTMs that predict fracture risk independently of BMD and capture dynamic skeletal processes that static densitometry cannot [22]. Our finding that higher CTX is independently linked to lower TBS supports the concept that high-turnover states may preferentially damage trabecular connectivity and microarchitecture, which in turn contributes to fracture risk in PLWH. At the same time, the relatively small incremental gain in AUC after adding CTX to a model already containing TBS echoes general-population data, where BTMs add only modest discrimination beyond clinical risk factors and BMD/FRAX but may still be useful for risk refinement in borderline cases and for monitoring treatment response [20,21,22]. Together, these observations support an integrated strategy in PLWH that combines early HIV diagnosis and rapid ART initiation to avoid profound CD4+ nadirs, optimization of body weight and lifestyle factors, avoidance of unnecessary TDF exposure, and targeted use of DXA, TBS, QUS and BTMs to identify those most likely to benefit from pharmacologic osteoporosis therapy [23,24,25,26,27].

From a therapeutic perspective, tenofovir alafenamide (TAF) is widely considered a more bone-sparing alternative to tenofovir disoproxil fumarate (TDF), with multiple switch studies demonstrating improvements in bone mineral density and bone turnover dynamics after replacing TDF with TAF. In this context, the observed association between prior TDF exposure and poorer trabecular microarchitecture supports guideline-consistent strategies that prioritize TDF-sparing regimens—such as TAF-based backbones—among PLWH with elevated skeletal risk, particularly older individuals or those with evidence of microarchitectural impairment [28,29,30].

Clinically, fracture probability is most often operationalized using FRAX^®^, and trabecular bone score can be incorporated as a validated adjustment to FRAX probability (FRAX-TBS). However, FRAX-TBS was not evaluated in the present study, and therefore we cannot determine whether the observed incremental discrimination of prevalent vertebral fracture status would persist when benchmarked against FRAX-based probabilities. Our findings nonetheless support the conceptual rationale for integrating microarchitectural information in PLWH, in whom FRAX may underestimate fracture probability. Beyond FRAX-TBS, a growing ecosystem of bone quality tools is emerging, including radiofrequency echographic multispectrometry (REMS) and REMS-derived fragility score, as well as high-resolution peripheral quantitative computed tomography and quantitative computed tomography-based approaches. These technologies may complement DXA/TBS in selected settings, but require validation in PLWH and in resource-diverse environments [31].

### 4.2. Study Limitations

This study has several limitations. Its cross-sectional design precludes inference about causality or temporal relationships between HIV-related factors, TBS changes and fracture occurrence. The sample size was modest (n = 87) and derived from a single tertiary HIV center, limiting statistical power for subgroup analyses and generalizability to other populations, including women, older patients and those with uncontrolled viremia. Participants were selected based on clinical indications for DXA, introducing potential referral bias toward higher-risk individuals. Vertebral fractures were assessed morphometrically at a single time point, and non-vertebral fractures were not systematically captured. Tenofovir exposure was classified as ever/never without detailed cumulative dose or duration. Important determinants of bone health, such as vitamin D status, gonadal function, physical activity and dietary intake, were not comprehensively measured, leaving residual confounding. Another key limitation is that FRAX^®^ probability, including FRAX adjusted for trabecular bone score (FRAX-TBS), was not calculated; thus, we could not benchmark our cross-sectional discrimination models against established fracture probability tools used in routine practice. In addition, we did not compare TBS with other imaging-based or emerging bone quality approaches (e.g., REMS-derived fragility score, HR-pQCT, QCT/opportunistic CT-based assessment), which limits conclusions regarding the relative performance of different technologies. Important determinants of skeletal health—such as vitamin D status, gonadal function, physical activity, nutritional intake, alcohol exposure, inflammatory comorbidities, and cumulative duration/dose of tenofovir exposure—were not comprehensively captured and may have contributed to residual confounding

## 5. Conclusions

In this cohort of ART-treated PLWH, bone fragility was highly prevalent, with 62% exhibiting low BMD, 37% degraded TBS and 17% morphometric vertebral fractures. Degraded TBS was independently linked to older age and more profound historical immunosuppression, and showed a strong association with tenofovir exposure. Importantly, TBS provided complementary information to DXA, significantly enhancing vertebral fracture risk discrimination beyond clinical variables and lumbar spine T-score alone. These data support integrating TBS into routine bone health evaluation for PLWH at risk of osteoporosis, and suggest that combined assessment with QUS and bone turnover markers may help define distinct skeletal phenotypes. Exploratory clustering suggested multidimensional bone phenotypes associated with differing prevalence of morphometric vertebral fractures; however, these patterns require validation before any clinical implementation.

## Figures and Tables

**Table 1:** Baseline demographic and HIV-related characteristics by trabecular bone score (TBS) category.

Variable	Total (n = 87)	Normal TBS (n = 25)	Partially Degraded TBS (n = 30)	Degraded TBS (n = 32)	*p*-Value
Age, years, mean ± SD	44.8 ± 8.9	39.7 ± 7.4	44.6 ± 8.6	49.1 ± 8.7	0.002
Female sex, n (%)	27 (31)	5 (20)	10 (33)	12 (38)	0.19
BMI, kg/m^2^, mean ± SD	25.1 ± 3.4	26.1 ± 3.2	25.3 ± 3.1	24.2 ± 3.6	0.089
Duration of HIV, years, mean ± SD	10.9 ± 5.0	8.8 ± 4.1	11.1 ± 4.8	12.7 ± 5.2	0.021
Nadir CD4+, cells/mm^3^, mean ± SD	252.3 ± 118.7	294.7 ± 123.1	258.9 ± 110.6	217.6 ± 107.4	0.035
Current CD4+, cells/mm^3^, mean ± SD	567.4 ± 176.3	613.7 ± 164.8	571.2 ± 173.6	529.3 ± 181.7	0.066
Viral suppression (<50 copies/mL), n (%)	68 (78)	23 (92)	24 (80)	21 (66)	0.041
Tenofovir DF exposure ever, n (%)	54 (62)	13 (52)	20 (67)	21 (66)	0.039
Current smoker, n (%)	29 (33)	7 (28)	9 (30)	13 (41)	0.27

Abbreviations: TBS, trabecular bone score; BMI, body mass index; HIV, human immunodeficiency virus; CD4+, cluster of differentiation 4; SD, standard deviation; n, number.

**Table 2:** DXA BMD, trabecular bone score, calcaneal QUS, and bone turnover markers by BMD status.

Measure	All Patients (n = 87)	Normal BMD (n = 33)	Low BMD * (n = 54)	*p*-Value †
Lumbar spine T-score, mean ± SD	−1.2 ± 0.9	−0.4 ± 0.7	−1.7 ± 0.8	<0.001
Total hip T-score, mean ± SD	−0.9 ± 0.8	−0.2 ± 0.6	−1.3 ± 0.7	<0.001
Femoral neck T-score, mean ± SD	−1.1 ± 0.8	−0.3 ± 0.6	−1.6 ± 0.7	<0.001
TBS (L1–L4), mean ± SD	1.28 ± 0.09	1.34 ± 0.07	1.24 ± 0.08	<0.001
Calcaneal SOS, m/s, mean ± SD	1543.7 ± 39.2	1561.3 ± 36.4	1532.4 ± 37.1	0.004
Calcaneal BUA, dB/MHz, mean ± SD	108.7 ± 12.4	112.6 ± 11.3	106.2 ± 12.7	0.018
Calcaneal BUA, dB/MHz, mean ± SD	108.7 ± 12.4	112.6 ± 11.3	106.2 ± 12.7	0.018

* Low BMD = osteopenia or osteoporosis based on lowest T-score. † *p*-values from independent-samples *t*-tests between normal and low BMD groups; Abbreviations: BMD, bone mineral density; DXA, dual-energy X-ray absorptiometry; TBS, trabecular bone score; SOS, speed of sound; BUA, broadband ultrasound attenuation; QUS, quantitative ultrasound; SD, standard deviation.

**Table 3:** Cross-classification of BMD and TBS categories and prevalence of vertebral fractures.

BMD Category	n	TBS Normal, n (%)	TBS Partially Degraded, n (%)	TBS Degraded, n (%)	Vertebral Fracture, n (%)
Normal BMD	33	18 (55)	9 (27)	6 (18)	3 (9)
Osteopenia	38	7 (18)	14 (37)	17 (45)	6 (16)
Osteoporosis	16	0 (0)	7 (44)	9 (56)	6 (38)
Total	87	25 (29)	30 (34)	32 (37)	15 (17)

Overall *p*-value for TBS distribution across BMD categories (chi-square): 0.002. Overall *p*-value for vertebral fracture prevalence across BMD categories (chi-square): 0.013; Abbreviations: BMD, bone mineral density; TBS, trabecular bone score; n, number.

**Table 4:** Bone quality parameters according to tenofovir disoproxil fumarate (TDF) exposure.

Variable	TDF Ever (n = 54)	TDF Never (n = 33)	*p*-Value
Age, years, mean ± SD	45.3 ± 9.1	44.0 ± 8.6	0.547
Lumbar spine T-score, mean ± SD	−1.4 ± 0.9	−0.9 ± 0.8	0.016
Total hip T-score, mean ± SD	−1.0 ± 0.8	−0.7 ± 0.7	0.071
TBS (L1–L4), mean ± SD	1.25 ± 0.08	1.32 ± 0.07	0.004
Calcaneal SOS, m/s, mean ± SD	1539.2 ± 38.7	1550.8 ± 38.1	0.083
Degraded TBS, n (%)	25 (46)	7 (21)	0.012
Vertebral fracture, n (%)	12 (22)	3 (9)	0.089

Abbreviations: TDF, tenofovir disoproxil fumarate; TBS, trabecular bone score; SOS, speed of sound; SD, standard deviation; n, number.

**Table 5:** Correlations between trabecular bone score (TBS) and selected clinical and biochemical variables (n = 87).

Predictor Variable	Pearson r	*p*-Value
Age, years	−0.41	<0.001
BMI, kg/m^2^	0.18	0.092
Duration of HIV, years	−0.29	0.008
Nadir CD4+, cells/mm^3^	0.27	0.012
Lumbar spine T-score	0.56	<0.001

Abbreviations: TBS, trabecular bone score; BMI, body mass index; HIV, human immunodeficiency virus; CD4+, cluster of differentiation 4; r, Pearson correlation coefficient.

**Table 6:** Multivariable logistic regression for predictors of degraded TBS (vs. normal or partially degraded TBS).

Predictor	Adjusted OR	95% CI	*p*-Value
Age (per 10-year increase)	1.78	1.13–2.83	0.013
BMI (per 1 kg/m^2^ increase)	0.91	0.83–0.99	0.031
Duration of HIV (per 5-year increase)	1.36	1.02–1.82	0.036
Nadir CD4+ < 200 vs. ≥200 cells/mm^3^	2.29	1.06–4.97	0.035
TDF exposure ever (yes vs. no)	2.14	0.97–4.72	0.059
Viral suppression absent vs. present	1.76	0.81–3.85	0.149

Model χ^2^ (6 df) = 18.7, *p* = 0.004; Nagelkerke R^2^ = 0.29; Abbreviations: TBS, trabecular bone score; OR, odds ratio; CI, confidence interval; BMI, body mass index; HIV, human immunodeficiency virus; CD4+, cluster of differentiation 4; TDF, tenofovir disoproxil fumarate; df, degrees of freedom.

**Table 7:** Determinants of Trabecular Bone Score (TBS) in Multivariable Linear Regression.

Predictor	Unit/Coding	B (Unstandardized)	SE	Standardized β	95% CI for B	*p*-Value
Age (per 10-year increase)	years	−0.024	0.008	−0.31	−0.040 to −0.009	0.004
BMI (per 1 kg/m^2^ increase)	kg/m^2^	0.006	0.003	0.19	0.000 to 0.011	0.038
Duration of HIV (per 5-year increase)	years	−0.011	0.005	−0.21	−0.022 to −0.001	0.034
Nadir CD4+ (per 100 cells/mm^3^)	cells/mm^3^	0.014	0.006	0.23	0.003 to 0.025	0.015
TDF exposure ever	yes = 1, no = 0	−0.031	0.013	−0.22	−0.056 to −0.006	0.017
Age × TDF interaction	per 10 years, TDF yes vs. no	−0.009	0.005	−0.16	−0.019 to 0.001	0.082

Outcome: TBS (L1–L4), continuous. All predictors entered simultaneously, including an age × TDF interaction term; Model statistics: R^2^ = 0.46; adjusted R^2^ = 0.41; F (7.79) = 9.6; *p* < 0.001; Abbreviations: TBS, trabecular bone score; B, unstandardized regression coefficient; SE, standard error; β, standardized regression coefficient; CI, confidence interval; BMI, body mass index; HIV, human immunodeficiency virus; CD4+, cluster of differentiation 4; TDF, tenofovir disoproxil fumarate.

**Table 8:** ROC and reclassification metrics for discrimination of prevalent morphometric vertebral fractures.

Model	Variables Included	AUC	95% CI	*p*-Value vs. AUC = 0.5	ΔAUC vs. Previous Model	*p*-Value for ΔAUC	Category-Free NRI vs. Previous (95% CI)	*p*-Value for NRI
A	Age, sex, BMI, smoking, duration of HIV	0.71	0.58–0.84	0.006	–	–	–	–
B	Model A + lumbar spine T-score	0.79	0.68–0.90	<0.001	+0.08	0.041	0.17 (0.02–0.32)	0.028
C	Model B + TBS	0.85	0.76–0.94	<0.001	+0.06	0.031	0.21 (0.04–0.37)	0.017

Outcome: morphometric vertebral fracture (yes/no). Models built sequentially; Abbreviations: AUC, area under the receiver operating characteristic curve; CI, confidence interval; BMI, body mass index; HIV, human immunodeficiency virus; TBS, trabecular bone score; NRI, net reclassification improvement.

**Table 9:** Cluster-Derived Skeletal Phenotypes Based on BMD, TBS, QUS, and Turnover Markers.

Characteristic	Cluster 1: “Low BMD/High Turnover” (n = 30)	Cluster 2: “Intermediate BMD/Mixed Turnover” (n = 27)	Cluster 3: “Near-Normal Bone” (n = 30)	*p*-Value
Lumbar spine T-score, mean ± SD	−1.9 ± 0.7	−1.1 ± 0.6	−0.4 ± 0.5	<0.001
TBS (L1–L4), mean ± SD	1.21 ± 0.07	1.27 ± 0.07	1.35 ± 0.06	<0.001
Calcaneal SOS, m/s, mean ± SD	1521.6 ± 32.9	1541.7 ± 33.8	1563.2 ± 34.4	<0.001
TDF exposure ever, n (%)	25 (83.3)	17 (63.0)	12 (40.0)	0.004
Vertebral fracture, n (%)	12 (40.0)	5 (18.5)	1 (3.3)	<0.001
Age, years, mean ± SD	47.8 ± 8.4	44.1 ± 8.7	42.1 ± 8.3	0.021

K-means clustering (k = 3) using lumbar spine T-score, TBS, calcaneal SOS, P1NP, and CTX. Abbreviations: BMD, bone mineral density; TBS, trabecular bone score; SOS, speed of sound; TDF, tenofovir disoproxil fumarate; SD, standard deviation; n, number.

## Data Availability

The data presented in this study are available on request from the corresponding author.

## Abbreviations

ART, antiretroviral therapy; AUC, area under the curve; BMD, bone mineral density; BMI, body mass index; BUA, broadband ultrasound attenuation; CI, confidence interval; CTX, C-terminal telopeptide of type I collagen; DXA, dual-energy X-ray absorptiometry; FRAX^®^, Fracture Risk Assessment Tool; HIV, human immunodeficiency virus; NRI, net reclassification improvement; OR, odds ratio; PCA, principal component analysis; P1NP, procollagen type 1 N-terminal propeptide; PLWH, people living with HIV; QUS, quantitative ultrasound; ROC, receiver operating characteristic; SD, standard deviation; SOS, speed of sound; TAF, tenofovir alafenamide; TBS, trabecular bone score; TDF, tenofovir disoproxil fumarate; VFA, vertebral fracture assessment.
